# Physiological and Biochemical Analysis Revealing the Key Factors Influencing 2-Phenylethanol and Benzyl Alcohol Production in Crabapple Flowers

**DOI:** 10.3390/plants13050631

**Published:** 2024-02-25

**Authors:** Qin Peng, Wenkai Tao, Fangyuan Yu, Qinqin Xiong, Chunshi Nong, Wangxiang Zhang, Junjun Fan

**Affiliations:** 1 College of Forestry, Nanjing Forestry University, No. 159 Longpan Road, Xuanwu District, Nanjing 210037, China; penqin@njfu.edu.cn (Q.P.); fyyu@njfu.edu.cn (F.Y.); ncs@njfu.com.cn (C.N.); 2 College of Horticulture, Jinling Institute of Technology, No. 99 Hongjing Avenue, Jiangning District, Nanjing 211169, China

**Keywords:** crabapple, 2-phenylethanol, benzyl alcohol, enzyme, C/N

## Abstract

Floral scent (FS) plays a crucial role in the ecological functions and industrial applications of plants. However, the physiological and metabolic mechanisms underlying FS formation remain inadequately explored. Our investigation focused on elucidating the differential formation mechanisms of 2-phenylethanol (2-PE) and benzyl alcohol (BA) by examining seven related enzyme concentrations and the content of soluble sugar, soluble proteins, carbon (C) and nitrogen (N), as well as the C/N ratio. The findings revealed that the peak content of 2-PE in *M.* ‘Praire Rose’ and BA in *M.* ‘Lollipop’ occurred during the end flowering stage (S4) and flowering stage (S3) periods, respectively. The enzyme concentration change trends of phenylpyruvate decarboxylase (PDL), phenylacetaldehyde reductase (PAR), soluble protein, C, N, and C/N ratio changes during the S3-S4 period in *M.* ‘Praire Rose’ and *M.* ‘Lollipop’ were entirely opposite. Correlation and PCA analysis demonstrated that the content of CYP79D73 (a P450) and N, and the C/N ratio were key factors in 2-PE production in *M.* ‘Praire Rose’. The production of BA in *M.* ‘Lollipop’ was more influenced by the content of phenylacetaldehyde synthase (PAAS), CYP79D73, and soluble sugar. As CYP79D73 exits oppositely in correlation to 2-PE (*M.* ‘Praire Rose’) and BA (*M.* ‘Lollipop’), it is hypothesized that CYP79D73 was postulated as the primary factor contributing to the observed differences of 2-PE (*M.* ‘Praire Rose’) and BA (*M.* ‘Lollipop’) formation. These results carry significant implications for crabapple aromatic flower breeding and the essential oil industry etc.

## 1. Introduction

Floral scents (FSs), comprising a diverse range of low molecular weight (30–300 amu) volatile compounds [1,2], are often heralded as the quintessence of flowers, embodying one of the three fundamental elements alongside shape and color. They serve as a critical criterion for assessing cut flowers [3]. Moreover, FSs play pivotal ecological roles, including the attraction of pollinators, and the enhancement of plant resistance [4,5]. The primary constituents of FSs encompass terpenes, phenylpropanoids/benzenes, and fatty acid derivatives [6]. In a previous study, the characteristic floral component of *Malus joensis* ‘Praire Rose’ was identified as 2-phenylethanol (2-PE) [7]. However, a distinct observation made by researchers found benzyl alcohol (BA) to be the characteristic component in 26 crabapple species and cultivars, with 2-PE detected in only one cultivar, namely *M.* ‘Jewelberry’ [8]. This intriguing phenomenon highlights the variability in floral components among crabapple species, adding a layer of complexity to the understanding of their unique scents. It has been observed that 2-PE serves as a key releaser for many plants, including roses [9], petunias [10], and *Magnolia champaca* [11], emitting a distinctive and alluring rose aroma [12]. Additionally, 2-PE has widespread application in various industries, contributing to the production of diverse flavors, fragrances, cosmetics, and food processing [13,14]. BA has been considered a pollinator-attractant in a diversity of pollination syndromes [15,16]. The synthesis of 2PE and BA is relevant across various industries and fields, from fragrance and flavoring to pharmaceuticals and chemical research, making it an important topic of study and exploration. Notably, 2-PE and BA are synthesized through the same precursor, L-phenylalanine (L-Phe). The synthesis of 2-PE involves two key steps: the conversion of L-Phe to phenylacetaldehyde (PALD), followed by the transformation of PALD into 2-PE [17,18]. While phenylacetaldehyde reductase (PAR) has been identified as the enzyme responsible for the second step in the synthesis of 2-PE, the first step displays notable variations among different plant species [18]. In *R.* ‘Hoh-Jun’ and *R. damascena* Mill., aromatic amino acid decarboxylase (AADC) has been identified as the enzyme that converts L-Phe to the intermediate compound PALD [9]. Conversely, in *Petunia hybrida* cv. Mitchell plants, phenylacetaldehyde synthase (PAAS) is the enzyme responsible for converting L-Phe [19]. Roses also employ a third enzyme called aromatic amino acid aminotransferase (AAAT), which catalyzes the formation of phenylpyruvic acid from L-Phe. This intermediate can then be further converted to PALD by phenylpyruvic acid decarboxylase (PDL) [20]. In the case of *Plumeria rubra*, the pathway for 2-PE production involves an intermediate (*E/Z*)-PAOx, which was catalyzed by a cytochrome P450 family 79D protein (PrCYP79D73) [21]. BA, a key component in the characteristic FS of common crabapple species and cultivars [8], is produced through phenylalanine ammonia-lyase (PAL). PAL also catalyzes the deamidation of L-Phe as part of the overall process. Benzaldehyde, originating from cinnamic acid and a precursor to BA, is generated through both CoA-dependent β-oxidation and CoA-independent non-β-oxidation pathways in the petunia [22,23]. The enzyme BPBT (benzyl alcohol/phenylethanol benzoyltransferase) plays a crucial role in promoting the synthesis of benzyl benzoate along with phenethyl benzoate [24,25,26]. PAR in *Rosa* has been reported to reduce benzaldehyde to BA with only half the activity as compared to its role in the synthesis of 2-PE [27]. The metabolism of FSs in plants involves secondary pathways and is regulated by various factors, including environmental conditions (light, temperature etc.), as well as physiological states [28,29,30]. Soluble proteins, indispensable intracellular biomolecules, play a pivotal role in catalyzing biochemical reactions, providing structural support, facilitating transport, and participating in signaling, among other functions [31]. They are integral to maintaining normal biological functions and metabolic activities in plants. The content of soluble proteins significantly contributes to aroma production in plants, as exemplified in *Camellia sinensis* [32]. Metabolic pathways in plant cells utilize various raw materials, where soluble sugars (e.g., glucose and fructose) serve as energy and carbon sources. Organic acids, amino acids, and other small molecule compounds participate in enzyme-catalyzed reactions to synthesize complex organic compounds like lignin [33]. The carbon to nitrogen ratio (C/N) reflects the plant’s utilization of carbon and nitrogen. Some studies suggest that elevated nitrogen levels in the soil can enhance the synthesis of FSs to some extent [34], particularly for compounds derived from phenylalanine [35]. According to the carbon balance hypothesis, higher C/N ratios induce the metabolism of carbon-based secondary compounds, including phenolic compounds [36,37]. It was demonstrated in a meta-analysis that phenolic reductions in simulated nitrogen (N) deposition were positively correlated with the C/N ratio and soluble sugar concentrations in plants [38]. This may result in a decrease in aroma compound production in flowers, affecting the richness and quality of FSs. Therefore, maintaining an appropriate C/N ratio is crucial for the production and quality of FSs in plants. Researchers are actively exploring ways to enhance the production of volatile compounds in flowers by modulating the physiological characteristics of plants, aiming to improve both the quality and quantity of floral scents. For instance, it has been found that the volatile content of β-ocimene and linalool in *Lilium* ‘Siberia’ increased in the presence of exogenous glucose and fructose [39].

Crabapples (*Malus* spp.) are deciduous small trees, belonging to the genus *Malus* in the Rosaceae family, characterized by a fruit diameter of less than or equal to 5 cm [40]. Varieties such as *M.* ‘Praire Rose’ and *M.* ‘Lollipop’ showcase diverse flower shapes, boasting significant ornamental value. These cultivars making them popular choices for a wide range of garden landscaping applications [41].

In plant breeding, an excessive focus on flower shape and color has led to the gradual loss of aroma in many varieties. Recent years have witnessed a growing interest in research on aroma components during flower development in plants [42,43,44]. However, most of the current crabapple FS research focuses on the characterization of the aroma components, and the effects of the differences in enzyme activities and physiological characteristics between crabapple varieties on the mechanism of their aroma formation remain unclear. To investigate the mechanism of the aroma component formation during crabapple development, we selected *M.* ‘Praire Rose’ and *M.* ‘Lollipop’ as materials, which exhibit significant differences in 2-PE and BA. Our study involved the determination of relevant metabolic enzyme concentrations, the content of soluble sugar, soluble protein, carbon (C) and N, as well as the C/N ratio. The objective was to elucidate the distinctions in the formation of characteristic benzene volatile compounds (BA and 2-PE). This study endeavors to provide new perspectives on the study of FS in plants, and establish a theoretical basis for scent breeding, scent biosynthesis, and physiological ecology in crabapple.

## 2. Results

### 2.1. Analysis of Volatile Compounds

**Figure 1:**
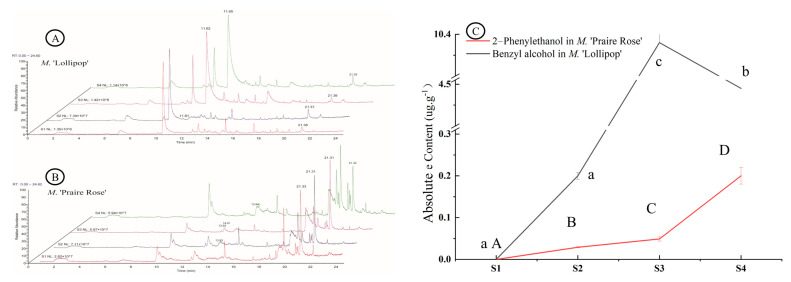
Chromatograms of GC/MS analyses (**A**,**B**) and changes in the absolute content of key volatile components in the two varieties (**C**). Note: the capital and small letter represent significant difference among flowering stages in *M.* ‘Praire Rose’ and *M.* ‘Lollipop’, respectively, (*p* < 0.05). The same as below.

Figure 1A,B showed that the volatile mass spectra of the two varieties exhibited pronounced differences in the positions and intensities of certain mass spectral peaks. This disparity indicated substantial distinctions in the compositions and structures of floral volatile compounds between the two varieties, showcasing notable inter-variety variations and relatively minor intra-variety differences. In *M*. ‘Lollipop’, BA emerges as a characteristic peak within the time period of 11.61–11.65 min, becoming a defining feature of this variety in the S3 and S4 stages. However, 2-PE was not observed in the four flowering periods (Figure 1A). In *M.* ‘Praire Rose’, 2-PE appeared in the time range of 13.84–14.01 min with a low peak intensity. BA was not detected (Figure 1B). Furthermore, the dynamics of BA in *M.* ‘Lollipop’ and 2-PE in *M.* ‘Praire Rose’ exhibit interesting temporal patterns. During the S1 period, neither BA in *M.* ‘Lollipop’ nor 2-PE in *M.* ‘Praire Rose’ were detected. Subsequently, the absolute content of BA experiences a noteworthy increase (*p* < 0.05), reaching a peak during the S3 period with a high absolute concentration, followed by a subsequent decrease. The absolute content 2-PE was relatively low in the developmental stages, although underwent a significant increase (*p* < 0.05) during the S4 period (Figure 1C).

### 2.2. Analysis of BA/2-PE Related Enzyme Concentrations

**Figure 2:**
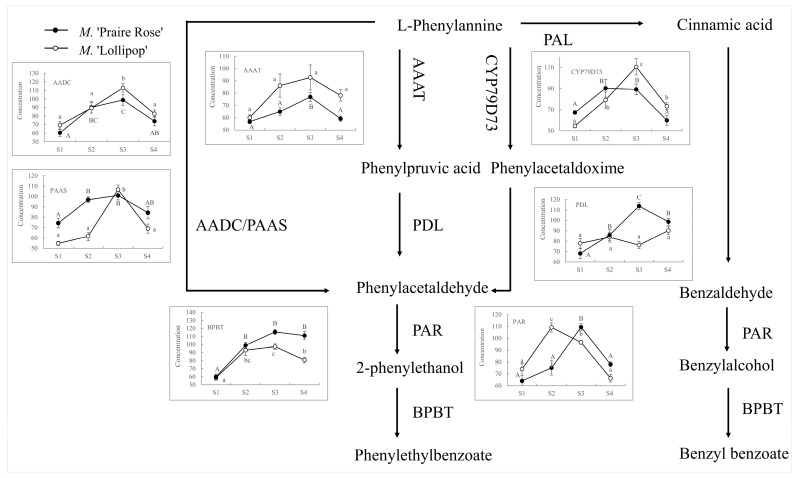
Analysis of key enzyme concentrations on BA and 2-PE-related metabolic pathways in four flowering stages of two crabapple species. Note: the capital and small letter represent significant difference among flowering stages in *M*. ‘Praire Rose’ and *M*. ‘Lollipop’, respectively. The same as below.

As shown in Figure 2, the enzymatic concentrations of AADC, PAAS, AAAT, CYP79D73, and BPBT displayed a similar pattern of variation across the four flowering stages for both *M.* ‘Lollipop’ and *M.* ‘Praire Rose’. The concentrations of AADC, PAAS, AAAT, BPBT, and CYP79D73 were observed to be at their lowest during the S1 stage, reaching their peak at the S3 stage, and then experiencing a significant decline at the S4 stage (*p* < 0.05). Comparative analysis between the S2 and S3 stages reveals that in *M.* ‘Lollipop’, the activities of PDL and PAR experienced a significant decrease (*p* < 0.05) during the S3 stage, while in *M.* ‘Praire Rose’, these concentrations saw a significant increase (*p* < 0.05) during the same stage.

### 2.3. Analysis of Other Physiological Indicators in Two Species

**Figure 3:**
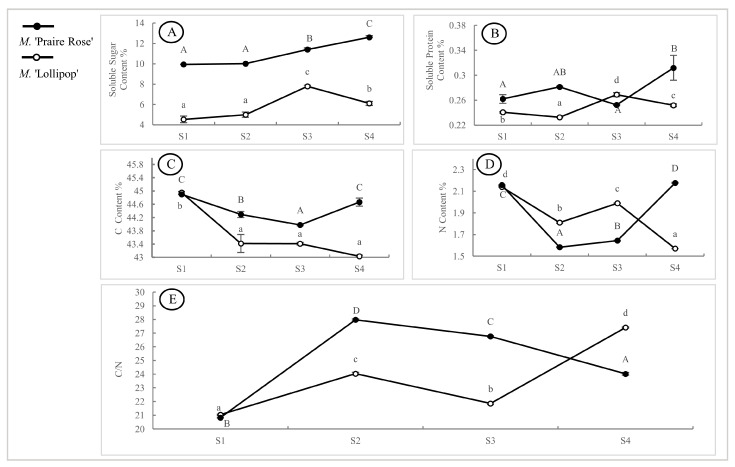
Analysis of content of soluble sugars (**A**), soluble proteins (**B**), C (**C**), N (**D**), and ratio of C/N (**E**) during four flowering stages in two varieties. Note: the capital and small letter represent significant difference among flowering stages in M. ‘Praire Rose’ and M. ‘Lollipop’, respectively, (*p* < 0.05).

Figure 3A showed that the soluble sugar content in the *M.* ‘Praire Rose’ flower had a significant increasing trend (*p* < 0.05) from the S2 to S4 stages, reaching its highest value at the S4 stage. Conversely, in *M.* ‘Lollipop’ flowers, the soluble sugar content increased significantly, peaking at the S3 stage and then decreasing significantly at the S4 stage (*p* < 0.05). The soluble sugar content of *M.* ‘Praire Rose’ was significantly higher than that in *M.* ‘Lollipop’ across all four flowering periods (*p* < 0.05). In Figure 3B, the soluble protein content of *M.* ‘Praire Rose’ demonstrated a dynamic pattern of increasing, decreasing, and then increasing again, reaching its highest level at the S4 stage. Conversely, *M.* ‘Lollipop’ displayed an opposite trend, decreasing, increasing, and then decreasing again, with the highest content at the S3 stage. The soluble protein content of *M.* ‘Praire Rose’ was only lower than that of *M.* ‘Lollipop’ in the S3 period, and significantly higher in the other three periods (*p* < 0.05). Figure 3C–E illustrated that the trends of C, N, and C/N ratios in *M.* ‘Praire Rose’ at the S1–S3 stages mirrored those of *M.* ‘Lollipop’, with an opposing trend observed in the S4 period. While there were no significant differences in C and N contents and C/N ratios at the S1 stage, *M.* ‘Praire Rose’ exhibited significantly higher C content at the S2–S4 stages. The N content was significantly lower in the S2 and S3 periods but significantly higher in the S4 period, compared to *M.* ‘Lollipop’. The C/N ratio demonstrated an opposite trend to the N content.

### 2.4. Correlation Analysis of Key Compounds with Physiological Indicators

**Figure 4:**
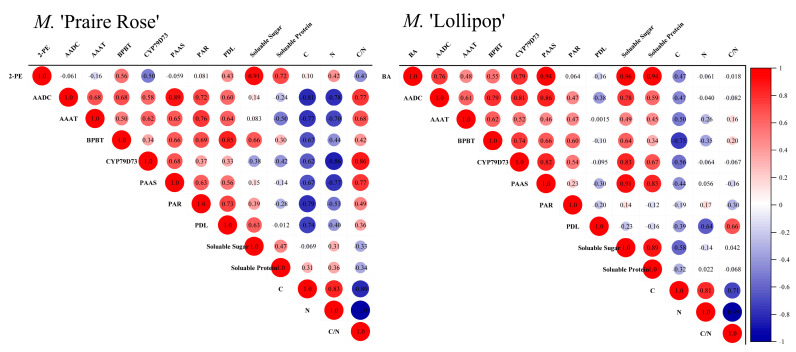
Correlation analysis between key volatile compounds and physiological metabolic indices in two varieties. Note: the correlation coefficient is given according to Pearson modulus.

Figure 4 showed the correlation analysis between two key volatile compounds (2-PE in *M.* ‘Praire Rose’ and BA in *M.* ‘Lollipop’) and physiological metabolic indices in the two varieties. For *M.* ‘Praire Rose’, the 2-PE content displayed strong positive correlations with the content of soluble sugar (0.91), soluble protein (0.72), and N (0.42), as well as the concentrations of BPBT (0.56) and PDL (0.56). It showed a negative correlation with the C/N ratio (−0.43) and the concentrations of CYP79D73 (−0.50). No significant correlations were observed with the C content and the concentrations of AADC, AAAT, PAAS, and PAR. All seven enzyme concentrations correlated negatively with the content of C and N, but positively correlated with the C/N ratio. For *M.* ‘Lollipop’, the BA content showed highly positive correlations with the concentrations of AADC (0.76), CYP79D73 (0.79), PAAS (0.94), AAAT (0.48), and BPBT (0.55), as well as the content of soluble sugars (0.96) and soluble proteins (0.94). However, the BA content showed negative correlation with the C content (−0.47) and showed no significant correlation with the concentrations of PAR and PDL, N content, and C/N ratio. The seven enzyme concentrations were mainly positively correlated with the content of soluble sugars and soluble proteins, negatively correlated with the C content, and not significantly correlated with the N content and the C/N ratio.

### 2.5. PCA Analysis of Key Compounds with Physiological Indicators

**Figure 5:**
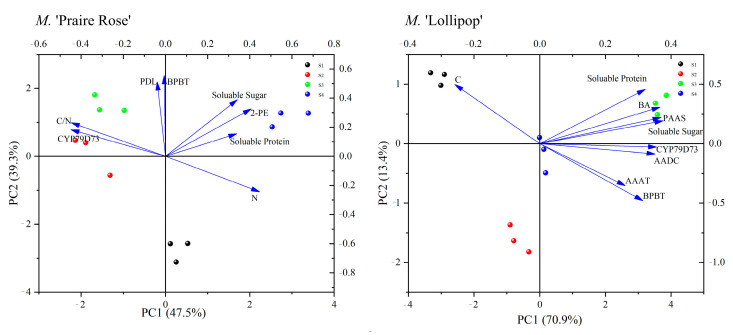
PCA analysis between key volatile compounds and physiological metabolic indices in two varieties.

In Figure 5, principal component analysis (PCA) was conducted based on the correlation analysis results, revealing a substantial total contribution exceeding 80% for both PC1 and PC2 in the two varieties. For *M.* ‘Praire Rose’, N exhibited a high positive score on PC1. The C/N ratio and the concentrations of CYP79D73 had high negative scores on PC1. For *M.* ‘Lollipop’, soluble sugars content and the concentrations of PAAS and CYP79D73 had high positive scores on PC1.

## 3. Discussion

This study investigated the dynamic variations in physiological and biochemical indices during the flower development stages of two crabapple species, with a specific emphasis on key volatile floral components: 2-PE in *M.* ‘Praire Rose’ and BA in *M.* ‘Lollipop’. The experimental results have unveiled compelling findings as CYP79D73 becomes the key regulator for the synthesis of BA, 2-PE, and N sources and the soluble sugar content becomes a key factor in 2-PE and BA synthesis, respectively.

### 3.1. 2-PE and BA Become the Key Differencial Compounds between M. ‘Praire Rose’ and M. ‘Lollipop’

The GC-MS results revealed significant differences in the types and relative abundance of volatile components among two crabapple species (*p* < 0.05), indicating a considerable diversity in aroma characteristics within the crabapple varieties. This observed variability in volatile compositions aligns with findings in other plants, such as tulip cultivars [45], *Osmanthus* [46], and *Lonicera japonica* [47]. The *M.* ‘Praire Rose’ flower was characterized by a particularly low content of 2-PE and the absence of BA, while *M.* ‘Lollipop’ exhibited a significantly high content of BA with no 2-PE detected. These distinct volatile profiles suggest the potential for differentiating crabapple varieties based on their specific aromatic compositions, a concept that has been explored in other plant species. For example, Zhang et al. suggested that releases such as BA and cinnamyl alcohol could differentiate intra-species varieties of *Prunus* from each other [48], and electronic noses were able to differentiate *Hedychium* based on odor intensity [49]. The differentiation of crabapple varieties based on volatile compounds of FS has yet to be further investigated, which is of great significance for plant classification and identification. The study also noted that the highest content of 2-PE in *M.* ‘Praire Rose’ and BA in *M.* ‘Lollipop’ primarily occurred in the S4 and S3 periods, respectively. It was observed in *Lonicera japonica* (*Caprifoliaceae*) that more odor is emitted at night than during the day to attract nocturnal moths [50]. This temporal variation in volatile release suggests a potential correlation with pollination and ecological functioning [51].

### 3.2. CYP79D73 Becomes the Key Regulator for the Synthesis of BA/2-PE in Two Species

AADC, PAAS, AAAT, CYP79D73, and BPBT are key enzymes in the biosynthetic pathway of 2-PE and BA in plants. The concentrations of these enzymes exhibited a consistent pattern of change in both plant varieties, aligning with the release pattern of BA in the *M.* ‘Lollipop’, reaching their peak during the S3 period. However, 2-PE was not detected during this phase. These results suggest that while the metabolic pathways of both varieties share similarities, there were also distinct differences. There is a possibility of synergistic effects among key enzymes in the biosynthesis of 2-PE and BA [52], but the specific mechanisms require further verification.

The absolute amount of 2-PE released from *M.* ‘Praire Rose’ peaked during the S4 period, deviating somewhat from the trend observed in the key enzyme concentrations. This observation suggests that the release of 2-PE in *M.* ‘Praire Rose’ flowers may have experienced a delay after its synthesis at S3, indicating susceptibility to various influencing factors [53]. Similar inconsistencies in volatile compound emissions from internal and external sources have been observed in other plant species such as *Jasminum* species [54] and *Chrysanthemum morifolium* [55]. Both 2-PE and BA biosynthesis utilize L-Phe as a substrate through different synthetic pathways, with some studies indicating a negative correlation between 2-PE and BA [56]. The enzyme CYP73D73 is known for its importance in benzene biosynthesis in *Prunus mume* plum [57], PDL is implicated in the production of 2-PE and indole-3-acetic acid (IAA) by the bacterium *Enterobacter* sp. [58], and PAR catalyzes the conversion of phenylacetaldehyde to 2-PE [59]. Knockdown of PAR has been demonstrated to prevent 2-PE biosynthesis [60]. In this study, the changes in PDL and PAR content exhibited complete opposition during the S2–S3 period in two varieties, with a significant decrease in *M.* ‘Lollipop’ and a notable increase in *M.* ‘Praire Rose’, surpassing that of *M.* ‘Lollipop’. Correlation analyses revealed a positive correlation between the content of 2-PE and the concentrations of BPBT and PDL, and a negative correlation with CYP79D73 concentration in *M.* ‘Praire Rose’. PCA analysis further confirmed that CYP79D73 was one of the key negative factors influencing 2-PE. These results indicated that PDL and PAR in the two varieties play differential role during the S2–S3 period, with PDL being associated with 2-PE synthesis but not a key factor influencing its production. The absolute content of BA showed a positive correlation with BPBT and CYP79D73 concentrations, with no significant correlation with PDL concentration. PCA analysis also confirmed that CYP79D73 was one of the positive key factors influencing BA. It is hypothesized that CYP79D73 was probably the key enzyme controlling the synthesis of carbon fluxes from BA/2-PE, and the concentrations could directly affect the differential volatilization of 2-PE and BA. The specific mechanisms of their differential functionality warrant further investigation.

### 3.3. N Sources and Soluable Sugar Content Become Key Factors Influencing in 2-PE and BA Synthesis, Respectively

Plants undergo photosynthesis, a process that converts carbon dioxide and water into soluble sugar molecules, particularly glucose. This process is a vital source of energy and raw materials for the synthesis of organic matter [61,62]. During the ripening of apple fruit, there is a gradual increase in soluble sugar content, coinciding with a significant accumulation of aroma volatiles [63]. The aroma of black tea or *Jasminum sambac* (L.) is also influenced by soluble sugars [64,65]. In this study, the dynamics of soluble sugars in *M.* ‘Praire Rose’ and *M.* ‘Lollipop’ closely paralleled the changes observed in the composition of 2-PE and BA, respectively, demonstrating strong positive correlations. The alterations in soluble protein, C, N, and the C/N ratio during the S3–S4 period exhibited opposing trends between the two varieties. These opposing variations in indices may signify substantial differences in nutrient uptake, metabolic pathways, or nutrient utilization during the S3–S4 stage in the two varieties. The existing literature indicates that the addition of N to N-deficient fermentation media enhances the release of 2-PE [66]. Similarly, applying foliar nitrogen during veraison in grapes significantly amplifies the volatile content of 2-PE in grape berries [67]. In this study, the level of 2-PE in *M.* ‘Praire Rose’ flowers exhibited a positive correlation with the content of soluble protein, soluble sugars, and N, while displaying a negative correlation with the C/N ratio. PCA analysis indicated that N and the C/N ratio were among the key factors influencing 2-PE. We posit that adequate N availability might contribute to heightened 2-PE levels. In *M.* ‘Lollipop’ flowers, BA demonstrated a strong positive correlation with soluble sugars and soluble proteins, and a negative correlation with the C content. PCA analysis indicated that soluble sugars were among the key factors influencing BA. This aligns with the trends observed in the corresponding indices, suggesting that sufficient soluble sugar content may boost BA content. This phenomenon could be attributed to plants evolving adaptive strategies over an extended evolutionary process, adjusting the synthesis of floral compounds to varying ecological conditions [68]. Therefore, our study’s hypothesis explored whether a proper increase in the N and soluble sugar could induce the release of 2-PE and BA, respectively, from these two species.

## 4. Materials and Methods

**Figure 6:**
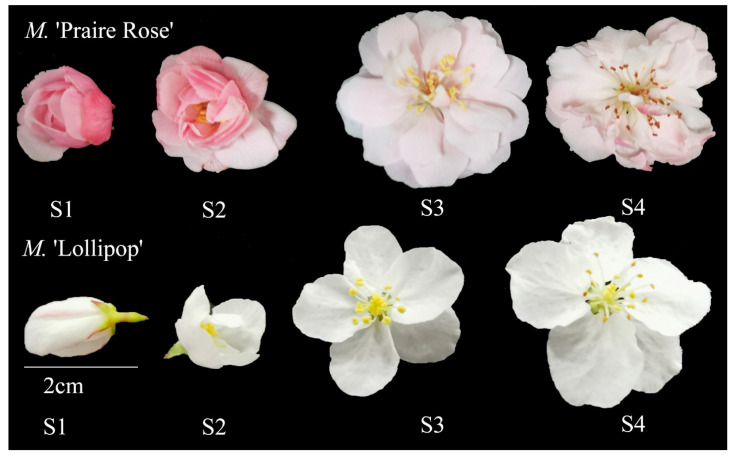
Experimental materials for this study. S1: large bud stage; S2: first flowering stage; S3: flowering stage; S4: end flowering stage.

The experimental site was in the National Crabapple Germplasm Resource Bank in Jiangdu District, Yangzhou City, Jiangsu, China, at coordinates 119°55′ E, 32°42′ N. The *M.* ‘Praire Rose’ and *M.* ‘Lollipop’, aged 3 and 4 years, respectively, were selected for the study (Figure 6). Each cultivar comprised five individual plants with similar growth characteristics. Flowers were collected at different developmental stages, including the large bud stage (S1), first flowering stage (S2), flowering stage (S3), and end flowering stage (S4). Fresh samples were used for GC-MS analysis and others were stored at −80 °C for enzyme concentrations and other physiological indices.

## 5. HS-SPME-GC-MS

Volatile compounds derived from each fresh sample flowers (4 g) were extracted using the headspace solid phase microextraction (HS-SPME) technique, conducted within a 100 mL beaker (sealed with cling film and 2.5 μL of methyl laurate (0.87 mg/mL methanol) added as an internal standard) immersed in a water bath at 50 °C for 30 min. The extraction process involved a polydimethylsiloxane-vinylbenzene (PDMS-DVB) extraction head, pre-activated at a high temperature of 270 °C for 0.5 h before the initial injection. The extraction depth, set at 2.5–3.0 cm, was performed at 50 °C for 30 min. A non-polar DB-5MS column (Agilent Technologies, Santa Clara, CA, USA), measuring 30 m in length, with an inner diameter of 0.25 mm, and a liquid film thickness of 0.25 µm, served as the analytical tool. High purity helium acted as the carrier gas, traversing the column at a flow rate of 1.0 mL/min via a non-split injection. The inlet temperature was maintained at 220 °C and the thermal analysis time was set at 5 min. The initial temperature was 45 °C, held for 1 min, ramped to 120 °C at a rate of 6 °C/min, held for 1 min, and then ramped to 220 °C at a rate of 10 °C/min. For mass spectrometry, the ion source temperature was set at 230 °C, and electron bombardment (EI+) ionization was employed with an electron energy of 70 eV. The detector voltage was adjusted to 1000 V, and mass spectrometry scanned over a range of 35–450 atomic mass units (amu) in full scan mode for 0.2 s. The transmission line temperature was set at 230 °C. The determination of linear retention indices involved the utilization of a series of n-alkane samples (C5–C30), and total ion currents were computed from peak areas, with the careful elimination of spurious peaks observed in the blank samples. Compound identification commenced with a comparison to the NIST11 library, with BA and 2-PE analyzed through a comparison with internal standards. The accurate calculation of the absolute content of VOCs was as follows [69]:

Ci = (Ai/As) × (ms/4)

where Ai and As represent the peak areas of the test compound and the internal standard substance, respectively; ms and m are the masses of the internal standard and sample, respectively.

## 6. Determination of Physiological Indices in Different Varieties

### 6.1. Determination of Key Enzyme Concentrations

The experiment was performed by double antibody sandwich ELISA [70]. The ELISA kit, provided by Nanjing Camilo Biological Engineering Co., Ltd. (Nanjing, China), is a typical sandwich enzyme-linked immunosorbent assay (ELISA) kit. Samples from the *M.* ‘Praire Rose’ and *M.* ‘Lollipop’ crabapple varieties at different stages (S1, S2, S3, and S4) were used as materials. The concentration of following enzymes associated with the metabolism of 2-PE and BA were measured: aromatic amino acid decarboxylase (AADC [71]), amino acid aminotransferase (AAAT [9]), benzyl alcohol/phenylethanol benzoyltransferase (BPBT [24]), CYP79D3 [24], phenylacetaldehyde synthase (PAAS [19]), phenylacetaldehyde reductase (PAR) [9], and phenylpyruvate decarboxylase (PDL [72]). For example, the pre-coated antibody is an anti-plant AADC monoclonal antibody. The detection phase antibody is a modified clonal antibody labelled with biocon (biotin). Samples and biotin-labelled antibodies were added sequentially to the wells of the plate and washed with PBS or TBS. TMB was used as a substrate for color development.

### 6.2. Determination of Soluble Sugar Content

The soluble sugar content was determined by the anthrone sulfate method [73]. A total of 0.1 g of dry sample was accurately weighed into a test tube, 8 mL of distilled water was added, and extracted in boiling water for 30 min. The extract was filtered into a 25 mL volumetric flask, the test tube and the residue were repeatedly rinsed with distilled water and fixed on the balance. A volume of 1 mL of the extract was taken and diluted 10 times. A total of 1 mL of the diluted extract was absorbed in a test tube and 5 mL of anthrone sulfate reagent was added. Color development occurred in boiling water for 10 min. The absorbance of the developed color was measured at the wavelength of 630 nm. The formula for soluble sugar content was as follows:

Soluble sugar content (%) = (C × VT × n)/(W × VS × 10^6^) × 100

where C: soluble sugar content from standard curve (ug); VT: total volume of extract (mL); VS: volume of sample extract taken for measurement (mL); n: dilution factor; W: sample quality (g).

The standard curve was plotted with the sugar content as the horizontal coordinate and the absorbance as the vertical coordinate, and the standard linear equation was solved as Y = 0.0048X + 0.0457, R2 = 0.9994. Three parallel experiments were performed for each sample.

### 6.3. Determination of Soluble Protein Content

Soluble protein content is determined by the Coomassie brilliant blue G-250 dye method [74]. A total of 0.1 g of dry sample was accurately weighed into a pre-cooled mortar. Then, 5 mL of pre-cooled phosphate buffer (0.1 mol. L^−1^, pH.7.0) was added several times to the mortar. The sample extract was prepared by centrifugation at 8000 rpm for 10 min (2–4 °C). A total of 0.9 mL of distilled water and 5 mL of Coomassie brilliant blue G-250 solution are added to 0.1 mL of the above soluble protein extract, mixed thoroughly, and allowed to stand for 2–3 min before colorimetry at the wavelength of 595 nm and calculation of soluble protein content. The formula was as follows:

Soluble protein content (%) = (C × VT × n)/(W × VS × 10^6^) × 100

where C: soluble protein content from standard curve (ug); VT: total volume of extract (mL); VS: volume of sample extract taken for measurement (mL); n: dilution factor; W: sample quality (g).

The standard curve was plotted with bovine serum protein content as the horizontal coordinate and absorbance as the vertical coordinate, and the standard linear equation Y = 0.0064X + 0.0677, R2 = 0.9995 was found. Three parallel tests were performed for each sample.

### 6.4. Determination of Carbon and Nitrogen Contents

Carbon (C) and nitrogen (N) contents were determined using an elemental analyzer (Supplied by Suzhou Elab Analytical Instrument Co., Ltd., Suzhou, China). Freeze-dried flower samples were ground using a liquid nitrogen grinder. The ground samples were then passed through a 300-mesh sieve. The resulting samples were placed in glass sample bottles for storage. Each sample was accurately weighed twice to approximately 3.000 mg (the PE AD6 model with a precision of 0.001 mg). The average was used as the final weighing result. Three copies were weighed separately, and the packaged samples were used to determine carbon and nitrogen contents using the elemental analyzer.

### 6.5. Data Analysis

All experiments in the study were repeated three times after collecting flower samples to ensure reliability of the results. SPSS Statistics 27.0 and Origin 2020 Pro software were used for graphical statistical analysis. Experimental results were expressed as mean ± standard deviation (SD). The Levene test was used to test for homogeneity of variances (*p* > 0.05). Differences among groups were statistically evaluated using one-way ANOVA followed by Duncan’s multiple comparison test. Significant differences between flowering stages were considered at the 5% probability level (*p* ≤ 0.05). Correlation and PCA analysis were performed based on Pearson modulus.

## 7. Conclusions

**Figure 7:**
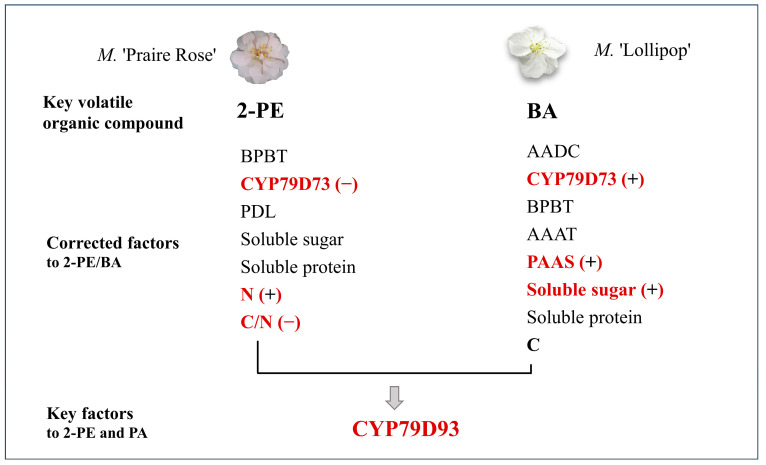
Several significant insights of *M.* ‘Praire Rose’ and *M.* ‘Lollipop’ flowers. Note: the red font indicates key factors to 2-PE/BA by PCA analysis; ‘+’ and ‘−’ represent the positive and negatively correlations, respectively.

In this study, a comprehensive analysis of physiological and biochemical parameters was conducted throughout four flower developmental stages, comparing two crabapple species with great differences in volatility. The primary focus was on understanding the variations and interrelations among 2-PE and BA, seven crucial metabolic enzymes participating in benzene synthesis pathways, and various biochemical factors including the content of soluble sugars, soluble proteins, C and N, and the C/N ratio. The findings of our investigation yielded several significant insights (Figure 7): (1) the release patterns of the key volatile components, 2-PE and BA, exhibited significant differences. (2) The activity trends of the five metabolic enzymes (AADC, PAAS, AAAT, CYP79D73, and BPBT) were consistent across the two varieties. However, during the S3-S4 period, the trends of PAR and PDL metabolic enzymes, as well as the soluble sugars, soluble proteins, C, N, and C/N ratio, were opposite. These disparities indicate significant differences in nutrient uptake, metabolic pathways, or growth requirements between the two varieties. (3) Correlation and PCA analyses revealed that the synthesis of 2-PE in *M.* ‘Praire Rose’ may have a greater dependence on N sources, while the synthesis of BA in *M.* ‘Lollipop’ may be more reliant on soluble sugar sources, and the concentrations of CYP79D73 may directly impact the volatile components of 2-PE and BA. Overall, these results contribute valuable insights into the physiological and biochemical characteristics of crabapple species. They serve as a foundational knowledge base, offering theoretical insights and practical guidance for the regulation of aroma components, species selection, and the development of related products within crabapple cultivation in the future.

## Data Availability

Data available on request from the authors.
